# Measuring a new facet of post traumatic growth: Development of a scale of physical post traumatic growth in men with prostate cancer

**DOI:** 10.1371/journal.pone.0195992

**Published:** 2018-04-27

**Authors:** Deirdre M. J. Walsh, Ann Marie Groarke, Todd G. Morrison, Garrett Durkan, Eamonn Rogers, Francis J. Sullivan

**Affiliations:** 1 School of Psychology, NUI Galway, Galway, Ireland; 2 University of Saskatchewan, Saskatoon, Canada; 3 National Cancer Control Programme, Saolta University Healthcare Group, Galway, Ireland; 4 Galway University Hospital, Galway, Ireland; 5 Prostate Cancer Institute, NUI Galway, Galway, Ireland; Harvard Medical School, UNITED STATES

## Abstract

**Purpose:**

This study developed a measure of physical post traumatic growth (physical post traumatic growth inventory; P-PTGI) in men with prostate cancer.

**Methods:**

A pool of items was created from themes identified in a qualitative study. A quantitative study was then conducted to assess the psychometric properties of the P-PTGI in a sample of 693 prostate cancer survivors.

**Results:**

Tests of dimensionality revealed that the 20-item P-PTGI contained two factors: Health Autonomy and Health Awareness. Results demonstrated that scale score reliability for the P-PTGI and its subscales was excellent. In support of the scale’s convergent validity, scores on the P-PTGI correlated positively with mindfulness and quality of life, and correlated negatively with depression and anxiety. A statistically significant correlation between the P-PTGI and another robust indicator of post traumatic growth attests to its concurrent validity.

**Conclusions:**

While further investigation of the P-PTGI’s psychometric properties is required, preliminary findings are promising.

## Introduction

Post traumatic growth (PTG) is defined as a collection of positive changes following a traumatic event. In the development of post traumatic growth, a traumatic event acts as a catalyst for the individual to re-evaluate his or her worldview. This can often result in distress but also various forms of positive growth [1]. Quantitative research in the area of PTG has revealed that, after a traumatic event, life changes often occur in five domains: namely, personal strength (or perceived changes in self); relationships with others; appreciation for life (or increased existential awareness); recognition of new possibilities; and spiritual change [1, 2]. Moreover, those who derive meaning from traumatic events also demonstrate better recovery and adjustment often leading to continued positive change and growth [3].

However, past research has not fully considered the distinct role that physical and health related traumas, such as cancer, may play in PTG [4]. The experience of physical trauma has elements unique from other traumas due to its internalized nature and direct impact on the body. Thus, it may contribute to a PTG experience that is distinct from the five dimensions currently posited by Tedeschi and Calhoun [1]. The existence of corporeally focused PTG has been suggested by recent research [5, 6, 7, 8] in which persons recovering from physical trauma may need to establish a new relationship with their bodies; change extant health behaviours; and embrace the “somatopsychic principle” wherein a stronger body helps to build a stronger mind.

There is growing recognition that current measurement tools do not adequately capture the full range of post traumatic responses [9] particularly in relation to positive outcomes that are facilitated through the experience of a *physical* trauma such as illness [10]. In addition, there are potential contributing factors to the growth process that have yet to be examined fully such as health status, level of physical activity and the role of the body. It is, therefore, important for researchers to investigate the broad range of physical and psychological benefits found in survivors exhibiting PTG [11, 12].

Previous attempts have been made to extend the main model of PTG [2]. Sabiston and colleagues [13], for example, articulated the first model of PTG which included other factors than those traditionally defined and involved in the growth process. Their findings, which emerged from a group of breast cancer survivors, revealed a unique relationship between physical self-perceptions and overall self-worth within the growth process [13]. The researchers presented a revised model of positive growth which included a focus on physical activity and personal control, as well as a shift in physical self-perceptions (e.g., strength and fitness).

This is an important avenue to explore as the corporeal nature of physical trauma may have wide implications for adjustment [6]. However, further research in this area is constrained as current measures of post traumatic growth do not include items which pertain to physical trauma or illness. As the work on this element of post traumatic growth has been exclusively qualitative, the current study aims to develop a quantitative measure of physical post traumatic growth in order to facilitate future research into physical PTG.

Furthermore, the relatively new concept of physical post traumatic growth has been exclusively investigated with females; in particular, women with breast cancer [7]. Examining PTG with prostate cancer survivors provides an opportunity to determine the extent to which older, male cancer survivors experience this phenomenon [14]. To the authors’ knowledge, physical post traumatic growth has yet to be examined with this sample.

## Method

A mixed method approach was used to develop a measure of physical PTG. This methodology was selected as the validity of psychological concepts in quantitative research may be enhanced when initially grounded in qualitative inquiry [15]. Qualitative methods are flexible enabling novel areas to emerge; ones that are not predicted in advance by the researcher. In the current study, this flexibility was important as no instrument assessing physical post traumatic growth currently exists. NUI Galway Research Ethics committee (12/MAY/10) and University Hospital Galway Research Ethics committee approved this study (12/DEC/11).

### Qualitative study phase

#### Participants

Participants were selected from databases of men who received surgery and adjuvant treatment at a University Hospital Rapid Access Prostate Cancer Diagnostic Clinic. Twenty-five individuals from each department (surgery or adjuvant treatment; 50 in total) were randomly selected. This allowed scope for non-responses to the request and for drop-outs while providing a sufficient sample for interview from the two different treatment groups. The final sample consisted of 18 men (9 per group) who had received treatment for prostate cancer at least one year prior to participating in the study. The average age of participants was 66.18 years (*SD* = 8.81, range = 52–82 years).

#### Procedure

The study protocol was approved by the Institutional University and the University Hospital Ethics Committee. Letters of invitation were sent to 50 potential participants. One follow-up phone call was made to each of the fifty invitees detailing the objectives of the study, and articulating key ethical issues in research with human participants (i.e., all information collected would be anonymous and confidential, and participant’s involvement could be terminated at any point without consequence). Written consent was obtained prior to participation. An interview guide of open-ended questions focusing on the individual’s prostate cancer experience was used flexibly to guide the interviews (see S1 File). The mean duration of each interview was 37 minutes (*SD* = 21.22). Recruitment continued until an adequate level of ‘saturation’ had been reached (i.e., additional interviews did not provide new insights; [16]).

#### Data analysis

The interviews were transcribed verbatim. A thematic analysis was conducted following the five phase framework outlined by Braun and Clarke [17]. The initial analyses were conducted after successive listening to the recordings and reading the transcripts. Notes were made in relation to significant comments made by each interviewee. To maintain the integrity of the data, memos were made throughout the analysis to create a paper trail of analytic decisions and theme formation. These were evaluated by two reviewers: an expert in psycho-oncology and an expert in qualitative methodology.

## Results

The main theme was entitled ‘New Awareness,’ which mapped onto P-PTG as described in previous reviews. A new awareness of health, which is often the by-product of physical trauma, contributed to new health behaviours such as health screening, visits to the doctor, improved nutrition and exercise. This ‘New Awareness’ encompassed three subthemes: 1) Renewed Appreciation of Health, 2) Engagement in New Health Behaviours, and 3) Acceptance of Age.

### Qualitative results: Main themes

#### Renewed appreciation of health

Many participants experienced a renewed appreciation of health. It was felt that there was now an opportunity to really value life: *“I was lucky…it’s good to get another chance to smell the flowers”* (Participant 1). This sense of appreciation also extended to a greater valuation of the physical self. Participants expressed how well their bodies had recovered following their treatment. For some, this greater appreciation was bittersweet. Many felt regret that they had taken their health for granted prior to the diagnosis and adjuvant treatment: “*I didn’t appreciate good health before I had any trouble*, *not as much anyway*. *Now I’d give a lot to be as good as I was before”* (Participant 2).

#### Engagement in new health behaviours

This greater appreciation of health was reflected in terms of participants’ desire to improve their health practices. Many participants felt they had been given an opportunity to assume more responsibility for personal health: “*It’s put that onus on me [to go for health checks]*. *It’s your body*, *and that’s what they are there for”* (Participant 9). Consequently, participants exhibited new health behaviours and made improvements to their diets: *“I’ve lost a stone and a half [21 lbs]…my style of eating has changed*. *I am now more healthy…we eat a lot of stir fries now than what we used to*. *I’m gone away from chips although I like them”* (Participant 8). Drinking and smoking behaviours also decreased to enhance health outcomes.

Visits to health professionals became routine and were used as a means of monitoring health: *“I go for these health checks once a year for all different diseases*. *You know because it is always there on your mind that I’m more susceptible because of my genetic make-up because I have had it [prostate cancer]”* (Participant 15). Participants also noted how physical check-ups did not seem important in the past, but were now regarded as an essential part of self-care: *“It’s all just part of life now”* (Participant 14).

#### Acceptance of age

Participants also noted that they were ‘at a certain age’ and expected their bodies to deteriorate. Therefore, the side effects of prostate cancer and/or treatment were seen as less problematic than they would be to younger men. Two quotations illustrate this point: *“If I was in my thirties and this happened*, *then I think it would be very different because it would be with you all your life*. *I know in less than 20 years’ time I’ll be coming towards the end anyway”* (Participant 9). *“Why the hell would I be mad at God*? *I’m 58 years of age*! *People haven’t lived a quarter of that time*, *half that time*!*”* (Participant 1).

### Item generation & refinement

The creation of items for the P-PTGI was grounded in scale development guidelines set forth by DeVellis [18]. All aspects related to physical post traumatic growth emerging from the qualitative interviews and extensive literature review were transformed into statements. Any acknowledgment of positive change following physical trauma was considered relevant to the exploration of the new construct of physical post traumatic growth and was included in the preliminary version of the P-PTGI.

Sixty-nine items were generated. All items were designed to enable expression of varying levels of growth following trauma and various levels of quality of life and experience of side effects. A 5-point Likert-type scale was used (-2 = greatly decreased; -1 = somewhat decreased; 0 = no change; +1 = somewhat increased; +2 = greatly increased). A ‘not applicable’ option was also included and treated as a missing value. Mean scores were computed using only those items regarded by participants as applicable to them, with higher scores denoting greater physical post traumatic growth.

To measure the content validity of the scale and ensure its readiness for psychometric evaluation, a panel of experts was recruited to assess the proposed items prior to participant recruitment. The role of the content experts was clearly defined using guidelines to assess the theoretical relevance of the items [19]. The panel consisted of content experts (i.e., individuals who had published in the fields of psychometrics [*n* = 1] and prostate cancer and post traumatic growth research [*n* = 4]). Experts were invited to be a part of the the panel based on their relevant publications and on-going research activity. Written informed consent was given prior to the expert panel review process was initiated. Experts were instructed to rate all items in terms of relevance to the construct [19]. Based on their feedback, 23 items were removed resulting in 46 items being retained for further testing. For example, one of the statements in the preliminary version was: “My pride in how my body has come through this crisis has… (greatly decreased/greatly increased).” One content expert recommended its removal because, as written, the item assumes that individuals had pride in their physical self prior to their diagnosis or, at least, *“awareness of it as an issue*.*”* Following discussion and further feedback, this item was deleted.

### Quantitative study phase

The purposes of this phase were to: 1) reduce the initial pool of scale items (*k* = 46) to a more manageable number; and 2) assess the dimensionality, scale score reliability and construct validity of the final version of the P-PTGI.

In relation to item reduction, the following criteria were applied.

First, high correlations between scale items may indicate redundancy; thus, if two items correlated with each other in excess of .90 [20], the item with the lower factor loading was deleted. Second, items were removed if inter-item correlations were weak (i.e., *r*s across other items within the factor were less than .30; [20]). Third, corrected item-total correlations for each factor were inspected; items with values less than .30 were removed [20]. Fourth, the minimal acceptable factor loading was .40, with no cross-loadings greater than .32 [21]. Any item which failed to meet these loading criteria was removed.

The dimensionality of the P-PTGI was assessed using principal axis factor (PAF) analysis, with oblique rotation. Cronbach’s alpha was used to gauge scale score reliability, with 95% confidence intervals being computed to determine lower- and upper-bound estimates for reliability. In terms of construct validity, four hypotheses were tested. First, mindfulness contains aspects of awareness and attention that are core parts of physical post traumatic growth [7,8]. Therefore, it was posited that participants obtaining higher scores on the P-PTGI also would evidence greater mindfulness (H1). Second, body awareness appears to be associated with physical post traumatic growth as illness may prompt greater general awareness in terms of listening to/monitoring one’s body [7,8]. Thus, it was predicted that participants higher in physical post traumatic growth, as measured by the P-PTGI, would also report greater body awareness (H2). Third, post traumatic growth (PTG) has been viewed as a form of cognitive adaptation in response to a cancer diagnosis that can give meaning and act as a buffer against distress (e.g., depression: [22]). Thus, it was hypothesized that participants’ scores on the P-PTGI would be negatively correlated with their scores on a measure of depression (H3). Fourth, Thornton and Perez [14] found that PTG was largely unrelated to quality of life (QoL) outcomes for prostate cancer survivors or partners. This non-association may be attributable to researchers’ use of the PTGI, which measures psychosocial areas of growth and may not capture the corporeal nature of illness and physical trauma [14,23]. In contrast, physical PTG, which denotes awareness and reconnection with the body post trauma, may be associated with greater QoL. Potentially higher PTG may lead to greater QoL as physical post traumatic growth has a greater focus on the body (i.e., greater appreciation of health and initiation of new health behaviours post trauma). Therefore, it was predicted that higher levels of physical post-traumatic growth, as measured by the P-PTGI, would be associated with higher levels of self-reported quality of life (H4). Finally, from a concurrent validation standpoint, physical post traumatic growth might be viewed as the ‘sixth’ dimension of post traumatic growth as defined by [1]. Therefore, it was hypothesised that participants higher in physical post traumatic growth also would be higher in traditional post traumatic growth (H5).

#### Participants

There were two phases of recruitment. In the first phase, a sample of 452 men attending a prostate clinic participated completing either an online (*n* = 360) or pen and paper (*n* = 92) survey. For the purpose of statistical analyses, the respondents were then randomly split into two samples: Data Set A (*n* = 226, age range = 46–89 years, *M* = 65.18, *SD* = 8.31) and Data Set B (*n* = 226, age range = 42–91 years, *M* = 65.22, *SD* = 8.30). To eliminate burden, only one questionnaire (the pool of candidate items for the P-PTGI) was administered.

In the second recruitment phase (henceforth known as Dataset C), 241 participants (age range = 44–88 years, *M* = 64.01, *SD* = 7.75) completed a shortened version of the P-PTGI along with validation measures. See Table 1 for further demographic information.

**Table 1:** Demographic information of Dataset A, B and C.

	*Dataset A* *(n = 226)*			*Dataset B* *(n = 226)*			*Dataset C* *(n = 241)*		
*Age*									
	*M =* 65.18	SD = 8.31	Range = 46-89yrs	*M* = 65.22	SD = 8.30	Range = 42-91yrs	*M* = 64.01	SD = 7.75	Range = 44-88yrs
*Time Since Diagnosis*		*N*	*%*		*N*	*%*		*N*	*%*
	< 3yrs	108	48.2		94	41.6		103	42.7
	3-4yrs	59	26.1		61	27.0		64	26.6
	5+ yrs	49	21.7		64	28.3		74	30.7
	Missing	9	4.0		7	3.1		0	0
*Type of Treatment*		*N*	*%*		*N*	*%*		*N*	*%*
	Surgery Only	54	23.9		73	32.3		53	22.0
	RT/BT	48	21.2		39	17.3		74	30.7
	HT	8	3.5		4	1.8		8	3.3
	Act. Sur	24	10.6		15	6.6		12	5.0
	Combination	81	35.8		79	35.0		80	33.2
	Other	7	3.1		12	5.3		13	5.4
	Missing	4	1.8		4	1.8		1	0.4
*Nationality*		*N*	*%*		*N*	*%*		*N*	*%*
	Europe	79	34.96		69	30.53		115	47.70
	N. American	137	60.62		140	61.94		113	46.90
	S. American	2	0.90		2	0.90		0	-
	Oceania	5	2.21		11	4.87		8	3.30
	Africa	0	-		1	0.40		2	0.80
	Asia/Middle East	3	1.33		3	1.33		2	0.80
	Missing	0	-		0	-		1	0.40

*Note*: *RT/BT =* radiotherapy/ brachytherapy; HT = hormone therapy; Act. Sur = Active Surveillance.

#### Validation measures (Dataset C)

Freiburg Mindfulness Inventory (FMI; [24]). The FMI is a 14-item scale that provides a brief measure of mindfulness. Responses are coded on a four point Likert scale from 1 (rarely) to 4 (almost always), with higher scores representing greater levels of mindfulness. The psychometric integrity of this measure has been demonstrated by Kohls and colleagues [25].

**Hospital and Anxiety Depression Scale** (HADS; [26]). The 14-item HADS provides a brief state measure of anxiety (HADS-A: 7 items; e.g., ‘I feel tense or wound up’) and depression (HADS-D: 7 items; e.g., ‘I still enjoy the things I used to enjoy’). Responses are coded on a four point Likert-type scale, with different response options for each question (e.g., 0 = not at all, 3 = most of the time; 0 = definitely as much, 3 = hardly at all). Higher scores denote greater anxiety or depression. Bjelland, Dahl, Haug, and Neckelmann’s [27] review of studies employing the HADS suggests that the scale possesses good scale score reliability and validity.

**Patient-oriented Prostate Utility Scale** (PORPUS; [28]). The PORPUS contains 10 questions: five are general (e.g., pain, energy, and relationship with physician) and five are prostate-cancer specific (e.g., sexual function/desire, and urinary frequency/ incontinence). Each item has four to six response options (e.g., in relation to the social support item: 1 = *Most of the time I feel supported by my spouse*, *family and/ or friends*; 4 = *Rarely feel supported by my spouse*, *family*, *and friends*), with higher scores denoting *lower* quality of life (QoL). In a recent psychometric assessment of prostate cancer-specific QoL instruments, the PORPUS was identified as one of the measures of choice [29].

**Post Traumatic Growth Inventory** (PTGI; [1]). The 21-item PTGI measures positive change following a trauma. The scale contains 5 dimensions: 1) relating to others (e.g., “I have a greater sense of closeness with others”); 2) new possibilities (e.g., “I developed new interests”); 3) personal strength (e.g., “I have a greater feeling of self-reliance”); 4) appreciation of life (e.g., “I have a greater appreciation for the value of my own life”); and 5) spiritual change (e.g., “I have a stronger religious faith”). Respondents indicate the degree to which the positive change has occurred in their life as a result of the trauma. Responses are coded on a 6 point Likert scale ranging from 0 (not at all) to 5 (a very great deal). Higher scores indicate greater levels of positive life change. Tedeschi and Calhoun [1] provide evidence attesting to the psychometric soundness of the PTGI.

**Private Body Consciousness Sub-Scale** (PBCS; [30]). The 5-item PBCS assesses body awareness which represents a disposition to focus on internal body sensations, being aware of interoceptive feedback, and being sensitive to changes in bodily states [30]. It uses a 5-point Likert scale (1 = extremely uncharacteristic; 5 = extremely characteristic), with higher scores denoting greater awareness. Research conducted by Skrinar and associates [31] as well as Christensen and colleagues [32] suggest that the PBCS is psychometrically robust.

#### Procedure

Ethical approval was obtained from the Institutional University Ethics Committee. National and international websites, forums or discussion groups were targeted for online recruitment from July 2013 to December 2013. Hard copy written consent was obtained for pen and paper questionnaires, while online participants completed a ‘tick box’ consent form in the same format as postal participants prior to study commencement. A moderator was identified and contacted with details of the study and a request that the link to the survey be posted. A website was also created which provided an information sheet stipulating that men who had prostate cancer within the past 10 years were eligible to participate; a link to the questionnaire; and contact details of the researcher. The purpose of the study and ethical requirements for research with human participants were described (i.e., participation was anonymous and voluntary). Minimum sample size was determined using guidelines for factor analysis (Fabrigar et al., 1999). It is generally regarded that a larger sample is better for this technique. The current studies have a sample-to-variable ratio of at least 4.9: 1 (for exploratory factor analysis). Given the strict criteria for item reduction outlined, the strength of the data is considered appropriate, and is therefore suitable for analyses.

### Quantitative results

#### Dimensionality of the P-PTGI

**Dataset A.** The factorability of the P-PTGI data was examined using Bartlett’s test of sphericity and the Kaiser-Meyer-Olkin (KMO) measure of sampling adequacy. For the KMO measure, values above .60 are necessary for EFA [33]. As Bartlett’s test was statistically significant (*p*< .001) and the KMO statistic exceeded .60 (KMO = .912), the data were suitable for EFA.

Forty-six items were subjected to principal axis factoring (PAF) with oblique rotation (direct oblimin, delta set at zero). Oblique rotation was employed as, should more than one factor emerge, some degree of interrelatedness among factors was expected.

Decisions regarding the number of factors to retain were based on a parallel analysis [34], in conjunction with an examination of the scree plot. Item reduction techniques as described above were applied.

A two-factor solution (eigenvalues = 14.25 and 5.07) consisting of 21 items (Health Autonomy: 10 items; Health Awareness: 11 items) was identified (see Table 2). The factor entitled Health Autonomy contains items pertaining to trust, confidence, strength, control, empowerment and a sense of achievement. The second factor, labelled Health Awareness, taps into a greater sense of physical awareness, greater attention and monitoring of the body and personal responsibility for health. Item loadings for Health Autonomy ranged from .42 to .90. For Health Awareness, loadings ranged from -.52 to -.85 (see Table 2). The correlation between the two factors was .40 suggesting they measure related, though distinct, aspects of the physical post traumatic growth experience.

**Table 2:** Factor loadings for principal axis factor analysis of the P-PTGI.

Item		Health Autonomy	Health Awareness
1	My trust in my body has…	.90	-.03
2	The feeling that I have control over my health has…	.82	-.07
3	My confidence in my body has…	.82	-.10
4	The empowerment I feel physically has…	.73	-.24
5	The control I feel over my body has…	.65	.02
6	The feeling that I have overcome any negative physical changes has…	.64	-.05
7	I feel the physical strength in my body has…	.53	.19
8	My confidence that my body will be strong enough to recover has…	.52	-.05
9	The feeling that my body is reliable has…	.46	.12
10	The sense of achievement in overcoming the physical obstacles of my illness has…	.42	-.20
11	My awareness of my own body has…	.01	-.85
12	The amount I listen to my body has…	-.08	-.79
13	The amount I monitor my body has…	-.01	-.76
14	My concern for my overall health has…	-.06	-.70
15	My responsibility for my health has…	.10	-.70
16	The attention I pay to how my body works has…	-.06	-.68
17	My awareness of parts of my body has…	-.01	-.60
18	The care I give my body has…	.20	-.58
19	My appreciation for good health has…	.04	-.58
20	The time I put into researching information about my health has…	-.05	-.56
**21**	**The respect I have for my health has…**	**.27**	**-.52**
Eigenvalues % of variance		14.25 33.14%	5.07 11.80%

*Note*: Health Autonomy (10 items), Health Awareness (11items). Items displayed have met item reduction criteria and are suitable for CFA. The item in bold was subsequently deleted resulting in a 20-item version.

**Dataset B.** The two-factor solution identified in Dataset A using PAF was tested in Dataset B with confirmatory factor analysis (CFA). As per Kline’s recommendations [35], model fit, or how adequately each item resides within a model [36], was assessed using multiple criteria: 1) absolute fit was examined using the chi-square/*df* ratio (*Q*) and the Root Mean Square Error of Approximation (RMSEA); and 2) comparative fit was assessed using Bentler’s comparative fit index (CFI). Stringent thresholds were employed: *Q* < 5, RMSEA < .08, CFI > .90 signify adequate fit while *Q* < 2, RMSEA < .06, and CFI > .95 denote excellent fit [33, 36].

The two factor model for the 21-item P-PTGI did not possess optimal fit when tested with dataset B: χ^2^ (188) = 458.99, *p* < .001; *Q* = 2.44; RMSEA = .08 (90% CI: .07-.09); CFI = .90. Thus, steps were taken to identify model misspecification. As part of this process, items were first thematically reviewed. Item 27 (‘The respect I have for my health has…’) was deemed to be thematically different from the other 10 items within the Health Awareness factor and, thus, was deleted. This had a significant positive effect on the model: χ^2^ (169) = 400.56, *p* < .001; *Q* = 2.37; RMSEA = .078 (90% CI: .07-.09); CFI = .91. Following the removal of item 27, five covariances were added to the model based on recommendations from modification indices, and justifiable on the grounds of closely related content (e.g., item 13: “The amount I listen to my body has…” and item 14: “The amount I monitor my body has…”). Fit statistics for all of the models tested are provided in Table 3.

**Table 3:** Confirmatory factor analysis models for P-PTGI (Dataset B).

Model	*x*^2^ (*df*)	*Q*	RMSEA (90% CI)	CFI	AIC	Δ AIC	*x*^2^*diff*	
							*x*^2^(*df*)	*P*
**Physical Post Traumatic Growth**								
P-PTGI 21 items	458.99 (188)	2.44	.80 (.071-.089)	.90	544.99	-	-	-
Delete item 21	400.56 (169)	2.37	.078 (.068-.088)	.91	482.56	62.43	58.43 (19)	.0005
Co-vary items 5 and 7	369.59 (168)	2.20	.073 (.063-.083)	.92	453.59	28.97	30.97 (1)	.0005
Co-vary items 12 and 13	345.30 (167)	2.07	.069 (.059-.079)	.93	431.30	22.29	24.29 (1)	.0005
Co-vary items 2 and 4	320.42 (166)	1.93	.064 (.054-.075)	.94	408.42	22.88	24.88 (1)	.0005
Co-vary items 16 and 19	305.58 (165)	1.85	.062 (.051-.072)	.94	395.58	12.84	14.84 (1)	.0005
Co-vary items 12 and 18	284.13 (164)	1.73	.057 (.046-.068)	.95	376.13	19.45	21.45 (1)	.0005
Final Model (5 covariances)	284.13 (164)	1.73	.057 (.046-.068)	.95	376.13	-	-	-

#### Scale score reliability of the P-PTGI

The 20-item version of the P-PTGI (see S2 File), resulting from the CFA, had excellent scale score reliability. For dataset A, Cronbach’s alpha coefficients were: total scale = .91 (95% CI = .88 - .92); Health Awareness subscale = .92 (95% CI = .91- .94); and Health Autonomy subscale = .91 (95% CI = .89-.93). For Dataset B, these values were: total scale = .92 (95% CI = .90 - .93); Health Awareness = .92 (95% CI = .90 - .93); and Health Autonomy = .91 (95% CI = .89 - .93). Finally, for Dataset C, Cronbach’s alpha coefficients were: total scale = .90 (95% CI = .88 - .92); Health Awareness subscale = .93 (95% CI = .91- .94); and Health Autonomy subscale = .92 (95% CI = .91-.94). Cronbach’s alpha coefficients, means and standard deviations, and score minima/maxima, for all measures, are provided in Table 4.

**Table 4:** Descriptive statistics for P-PTGI and validation measures.

Scale	M	SD	Cronbach’s Alpha (α)	95% CI	Possible Range	Attained Range
P-PTGI						
Dataset A	0.51	0.57	.91	.88 - .92	-2.00 - +2.00	-1.70 - +1.85
Dataset B	0.51	0.60	.92	.90 - .93	-2.00 - +2.00	-1.30 - +2.00
Dataset C	0.41	0.56	.90	.88 - .92	-2.00 - +2.00	-1.50 - +2.00
**Body Awareness**	17.01	4.08	.77	.72 - .81	5–25	5–25
**Depression**	4.05	3.36	.78	.74 - .82	0–28	0–16
**Mindfulness**	36.83	8.65	.91	.89 - .93	14–56	14–52
**PTG-Total**	38.37	26.64	.97	.96 - .97	0–105	0–105
**Quality of Life**	71.47	15.06	.69	.62 - .75	23.52–100	0–100

#### Scale score validity of the P-PTGI

As predicted, participants evidencing greater levels of physical post traumatic growth also reported greater mindfulness (H1; *r* = .19, *p* < .01) and quality of life (H4; *r* = .34, *p* < .01), and lower levels of depression (H3; *r* = -.30, *p* < .01). In contrast to our predictions, no statistically significant association was observed between physical post traumatic growth and body awareness (H2; *r* = 12, p = ns). As well, physical post traumatic growth, as measured by the P-PTGI, correlated positively with a measure of post traumatic growth (H5; *r* = .42, *p* < .01) suggesting that this scale possesses concurrent validity (see Table 5 for further details).

**Table 5:** Summary of inter-correlations for subscales and total P-PTGI, PTG, mindfulness and body awareness scores, HADS and QoL.

	Subscale	1	2	3	4	5	6	7	8	9	10	11	12	13	14
1	P-PTGI–Health Autonomy	-													
2	P-PTGI–Health Awareness	.19**	-												
3	P-PTGI–Total	.81***	.73***	-											
4	PTG–relating	.18**	.40***	.37***	-										
5	PTG–new possibilities	.19**	.40***	.37***	.78***	-									
6	PTG–personal strength	.32***	.42***	.47***	.81***	.83***	-								
7	PTG–spiritual change	.12	.27***	.24***	.64***	.66***	.63***	-							
8	PTG—appreciation	.13*	.53***	.41***	.73***	.75***	.73***	.57***	-						
9	PTG–Total	.22**	.46***	.42***	.94***	.92***	.92***	.75***	.84***	-					
10	Mindfulness	.18**	.12	.19**	.14*	.20**	.18**	.17**	.11	.18**	-				
11	Body Awareness	.02	.18**	.12	.11	12	.14*	.10	.08	.13*	.16*	-			
12	Anxiety	-.31**	-.01	-.22**	-.04	.01	-.03	-.03	-.01	-.02	-.46**	.04	-		
13	Depression	-.38**	-.06	-.30**	.00	.00	-.02	-.01	-.02	-.01	-.50**	.05	.58**	-	
14	Quality of life	.43**	.05	.34**	-.09	-.03	-.02	-.08	-.03	-.06	.31**	-.06	-.40**	-.55**	-

* = p < .05

** = p < .01

*** = p < .001

In order to assess incremental validity, squared semi-partial correlations were conducted to examine the relationship between P-PTG and outcomes of anxiety and depression and quality of life while controlling for PTG. Results indicate that controlling for PTG, P-PTG significantly accounts for an additional 16% of the variance in quality of life (r^2^ = .16), accounts for an additional 11% of the variance in depression (r^2^ = .11) and accounts for an additional 5% of the variance in anxiety (r^2^ = .05).

Further analyses were conducted to assess construct validity by including demographic and health-related factors (i.e., age, time since diagnosis, and treatment group), post-traumatic growth (total score), and physical post-traumatic growth subscales (health autonomy and health awareness) in a series of hierarchical regression analyses to determine their contribution to health outcomes of quality of life, depression and anxiety, respectively (see Table 6). Notably, the P-PTGI subscale of “Health Autonomy” was the strongest unique predictor for quality of life (r^2^ = .21), depression (r^2^ = .16), and anxiety (r^2^ = .11), while PTG-Total was significant for quality of life (r^2^ = .03) only. Of interest, the inclusion of PTG subscales (e.g., Relating to others), did not significantly alter the results of these regression analyses (results not shown, available from corresponding author). Thus, the P-PTGI subscales added explanatory power to the prediction of quality of life, depression and anxiety in men diagnosed with prostate cancer.

**Table 6:** Hierarchical linear regressions for demographics, post-traumatic growth (PTG), and physical-post traumatic growth subscales predicting quality of life, depression and anxiety.

Variables									
**Criterion Variable: Quality of Life**									
**Step 1**	t	β	Sig.	T	Β	Sig.	t	β	Sig.
Age	-1.76	-.11	.079	-1.78	-.11	.087	**-2.81**	**-.16**	**.005**
Time Since Treatment	1.25	.08	.212	1.18	.08	.240	1.38	.08	.170
Treatment Group	**-3.51**	**-.22**	**.001**	**-3.48**	**-.22**	**.001**	**-2.40**	**-.14**	**.018**
**Step 2**									
PTG-Total				-0.64	-.04	.523	**-2.58**	**-.17**	**-.010**
**Step 3**									
Health Autonomy							**7.66**	**.46**	**< .001**
Health Awareness							0.73	.05	.469
**Criterion Variable: Depression**									
**Step 1**									
Age	-0.74	-.05	.459	-0.75	-.05	.456	-0.07	-.00	.947
Time Since Treatment	-0.67	-.04	.502	-0.69	-.05	.488	-0.82	-.05	.415
Treatment Group	-0.44	.03	.660	0.45	.03	.653	-0.71	-.04	.480
**Step 2**									
PTG-Total				-0.27	-.02	.785	1.22	.08	.223
**Step 3**									
Health Autonomy							**-6.30**	**-.40**	**.000**
Health Awareness							-0.33	-.02	.740
**Criterion Variable: Anxiety**									
**Step 1**									
Age	-1.61	-.10	.108	-1.63	-.11	.104	-0.99	-.06	.320
Time Since Treatment	**-2.06**	**-.13**	**.040**	**-2.13**	**-.14**	**.035**	**-2.35**	**-.15**	**.020**
Treatment Group	-0.15	-.01	.879	-0.12	-.01	.902	-1.16	-.07	.249
**Step 2**									
PTG-Total				-0.75	-.05	.452	.06	.00	.953
**Step 3**									
Health Autonomy							**-5.09**	**-.33**	**< .001**
Health Awareness							0.71	.05	.479

Note: **bold** indicates statistical significance *p* < .05

## Discussion

This is the first exploratory study of physical post traumatic growth in a male sample with a physical illness. The current research adds incremental value in terms of its qualitative findings, but also in terms of addressing measurement issues in this burgeoning area. Specifically, the qualitative findings add new insight into perceptions of positive growth related to the body, expressed in a new awareness, with new health behaviours, an enhanced appreciation of the body and a new perspective on ageing. The qualitative analysis of changes related to the body following physical trauma supports existing research in breast cancer and provides additional understanding of the way physical trauma can act as a springboard to higher levels of functioning [5,6,7,8].

The exploratory qualitative findings are important as the experience of recovery in prostate cancer, especially in terms of PTG, has received scant attention. This dearth of research is of concern given qualitative findings that suggest levels of positive growth across various cohorts of breast cancer survivors [7]. Given that older cancer survivors, in comparison to their younger counterparts, may experience cancer differently and have a different perspective on existential awareness, research with an older cohort is needed.

Importantly, in the current study, the majority of participants cited their diagnosis as facilitating greater awareness of their “health potential.” These accounts support previous research that a new awareness and understanding of health can be cultivated following a health-related trauma [8]. This pattern of positive health behaviour change following illness has been discussed in terms of having the potential to be a teachable moment in a person’s life [37]. Previous studies have indicated how recommendations from medical and nursing staff strongly influence positive changes in lifestyle in men following prostate cancer [38]. Indeed, we found that many participants discussed new health and screening behaviours with enthusiasm. Participants also showed a heightened awareness of changes in, and appreciation for, their bodies. Similarly, Crane-Okada and colleagues [39] found that a reconnection with the body resulted in a heightened awareness of one’s corporeality and a greater connection to both positive and negative feelings post breast cancer.

The reports of positive change in this cohort of men is interesting as it contrasts with Thornton and Perez’s [14] finding that men with prostate cancer scored lower on PTG than previous cohorts of women with breast cancer. However, we contend that this low PTG is due to the lack of reference to the physical self in the post traumatic growth inventory that was used (i.e., PTGI). It must be acknowledged that the strongest correlation obtained between PPTGI and PTGI is .42. Thus, additional validation work is required. In the current study, it may be that the moderate correlation between the P-PTGI and the PTGI illustrates that the physical aspect of post traumatic growth, which was previously omitted from measurements of PTG, is a related but distinct component of post traumatic growth. The incremental value of including the physical component of PTG in the prediction of distress and quality of life highlights the importance of discrete aspects of growth. However, as this is the first measure of this potential additional dimension of PTG, further research is required to clarify these relationships.

The current findings support a previously proposed model of positive psychological growth following breast cancer [13]. The theme ‘New Awareness’ and subsequent P-PTGI factors ‘Health Autonomy’ and ‘Health Awareness’ reflect more physical aspects which have been under-represented in traditional conceptualisations of positive change and post traumatic growth. Sabiston and colleagues’ model [13] focused on positive change following trauma including physical perceptions such as: athletic identity, competitiveness, aerobic fitness, strength and physical competence. These areas highlight how illness provides a path to growth and an opportunity for individuals to consider their bodies in new ways. For instance, in the current study, strength and physical competence were a large part of the prostate cancer journey and goals for recovery. Given the emergence of the sub-theme entitled “Engagement in New Health Behaviours,” it is interesting how Bevan, Maxfield and Bultmann [40] suggest that with increasing proximity to mortality, adults over sixty significantly increase healthy behaviours in later life. This assertion supports how men with a prostate cancer diagnosis may engage in positive health behaviour changes. The current qualitative and quantitative work support the need for further exploration of how these ‘modifiable factors’ such as improved health behaviours, may play a role in the process of PTG [41].

However, further light cannot be shed on this issue due to the reliance on measures that do not make explicit reference to the physical self (e.g., the PTGI). By ignoring the dimension of physical post traumatic growth, current research on PTG and illness may be unable to fully capture the growth experience. This must be addressed as the majority of PTG research is conducted with groups who have experienced illness which would have potential implications for the role of the body. The development of the P-PTGI scale and its psychometric assessment constitute an initial effort in better understanding this complex issue.

The importance of identifying how physical post traumatic growth is related to potentially modifiable factors within the adjustment process such as mindfulness, depression, and quality of life can be seen in the current study. Given the positive results, whereby higher levels of physical post traumatic growth, as measured by the P-PTGI, were associated with greater levels of mindfulness, better perceived quality of life and lower levels of depression, this is an area that must be deemed a priority for chronic illness and survivorship interventions. Interestingly, in the current study, physical post traumatic growth was not correlated with body awareness, despite previous qualitative work suggesting that the two are connected [6,7,8]. Potentially, the measure of body awareness used may have influenced this null finding. This study used the PBCS, which is a subscale of the Body Consciousness Questionnaire (BAQ; [30]). This subscale aims to capture ‘private body consciousness’ or awareness of the body in non-affective states to allow differentiation between health anxiety and bodily awareness. However, given the exploratory nature of the current study, potentially the full BAQ should have been utilised including all three factors (i.e.,‘public body consciousness’, ‘private body consciousness’ and ‘body competence’; [30]). This lack of significance was unexpected and needs further investigation among different cohorts to determine whether it is an idiosyncratic or robust finding.

### Limitations of the current research

There are several limitations to consider in relation to this study. The time frame (between one and ten years post diagnosis) was selected as a participant inclusion criterion as previous longitudinal analyses of post traumatic growth have not yet conclusively established the presence of PTG further than ten years post trauma [42,43]. Given the current study’s inclusion criteria, levels of P-PTG reported by participants may not be reflective of the earliest development stages of physical post traumatic growth and likewise may not capture long term physical post traumatic growth (i.e., ten years post trauma).

The questionnaire also contains only items which have a positive focus, which has been a criticism of past PTG research. However, this issue was mitigated somewhat by our inclusion of response options such as “not applicable to me” and “no change” which, at the very least, enables participants to articulate the *absence* of positive change.

There are also some other limitations in relation to the dual postal and online recruitment strategy. Previous research has raised concerns regarding the validation of participant identities, especially with the online recruitment of a specific cohort (i.e., prostate cancer survivors). There are several ways to circumvent many issues in relation to participant identity, including confirming reports of demographic information at multiple time points or matching IP addresses to reported given address [44]. However, these strategies were not utilised for the current study as participant consent was not established to use data, such as IP addresses, for validation purposes. This can be considered for future recruitment drives. Indeed, electronic methods of data collection can limit the opportunity for face-to-face participant contact and the subsequent development of participant rapport [44], this may have hindered the disclosure of personal information in relation to post trauma experience, however qualitative methods were utilised with the scale development phase.

Finally, it must be acknowledged that this study constitutes the first attempt to capture and assess a physical component of post traumatic growth, therefore further research is required to clarify the relationships between physical post traumatic growth, the original post traumatic growth subscales, and health-related constructs (e.g., quality of life).

### Directions for future research

As yet, there is no comprehensive and cohesive framework for post traumatic growth which considers how the type of trauma may influence the growth trajectory and even the type of growth experienced. Although the current study delineates associations between physical post-traumatic growth and individual difference variables such as perceived quality of life and depression, there is a need to explore more complex inter-relationships. Structural equation modelling may be helpful in this regard. Future research is warranted to identify ways in which physical post traumatic growth might be facilitated or enhanced. Current research has tentatively suggested that engaging in physical activity may be a worthy avenue of further exploration [45]. Much is yet to be understood regarding the physical post traumatic growth experience which may differ in accordance with the type of health trauma experienced. As this is the first study to investigate this phenomenon with prostate cancer survivors, there is considerable work to be done in terms of establishing if this measure would be suitable for distribution to women (e.g., breast cancer survivors), younger adults and other trauma survivors. Indeed, the generalizability of this scale may be limited, and therefore, caution must be applied when interpreting the current findings. P-PTG construct and measurement urgently needs to be further explored in relation to other diagnoses, ages, and women.

### Conclusion

The experience of prostate cancer was found to have an impact on men’s embodied self-perceptions. Furthermore, the extent to which participants experienced a new awareness of the body indicates a distinct dimension of growth following physical trauma.

The qualitative study, demonstrating novel evidence in support of the concept of physical PTG, is an important step in understanding physical post traumatic growth in older adult males with prostate cancer, especially given the scant attention given to this group. Furthermore, measurement issues may compound research seeking to delineate relationships between post traumatic growth and outcomes following physical illness or trauma. Inclusion of a physical dimension of post traumatic growth is important when examining growth following illness, particularly with survivor groups of men with prostate cancer who may be experiencing ongoing side effects following treatment. The development of a psychometric measure of this additional facet of post traumatic growth, using a large sample and robust validation instruments, is an incremental step in acknowledging the importance of PTG in physical trauma and is imperative for advancing the needs of trauma survivors in terms of adjustment and well-being.

## Supporting information

S1 File Additional file 1. (DOCX)

S2 File Additional file 2. (DOCX)
