# Supplementary material for: Measuring a new facet of post traumatic growth: Development of a scale of physical post traumatic growth in men with prostate cancer
Source: PLoS One. 2018 Apr 27;13(4):e0195992. doi: 10.1371/journal.pone.0195992 (PMC5922578; doi:10.1371/journal.pone.0195992)
Supplement: S1 File — (DOCX) [file pone.0195992.s001.docx]

*Set of Guiding Questions and Probes Used to Facilitate Discussion of Relevant Topics.*

| 1. Introductions, confidentiality and freedom to withdraw at any time 2. Conversation recorded with their permission 3. Basic demographic questions 4. Could you tell me about your experience with prostate cancer?    - When did you first think you had prostate cancer?    - Were there any symptoms?    - Tell me about your diagnosis    - Tell me about your treatment 5. Which treatment did you choose?  - Why did you choose this treatment?  1. Can you tell me about how the treatment affected your body?  - What were the main side effects?  1. How did the side effects impact on you?  - What happened? - How did you manage/cope?  1. How did you feel during your treatment? 2. How did you feel when your treatment was complete? 3. After your diagnosis and treatment, did you think about your body differently?  - In what way did you think about it?  1. Do you think your body has changed?  - In what ways?  1. Do you think having prostate cancer changed you in any way?  - In what ways?  1. Have you changed how you treat your body?  - In what ways?  1. How do you see yourself in the future? 2. Is there anything positive that came from your experience? 3. De-briefing, contact number given in case of questions, support services mentioned also. |
| --- |
| ***Probes used throughout the interview:***   - Could you tell me more about that? - How come? - Why? - Could you tell me what you were thinking at the time? - What do you mean by that? - How did you feel about that? |
